# Majestic palm trees: tropical nature and imagination in the context of nineteenth century European imperialism

**DOI:** 10.1590/S0104-59702025000100042en

**Published:** 2025-09-22

**Authors:** Alessandra El Far

**Affiliations:** i Associate professor,Department of Social Sciences/Universidade Federal de São Paulo. Guarulhos – SP – Brazil el.far@unifesp.br

**Keywords:** Palm tree, Tropicality, Representation, Imperialism, Nineteenth century

## Abstract

This article analyzes how palm trees were represented in the nineteenth century as majesties of tropical nature by European botanists and horticulturists amid an imagination of colonial domination. To this end, I will examine texts and images in horticultural publications, printed in England, France, Belgium and Portugal, which repeatedly conveyed the idea of the tropics as a place governed by its nature, in which the palm tree gained centrality, in contrast to Europe, taken as a model of civilization. It was in this context that the sumptuous palm houses came to be constructed, which also functioned as privileged symbols of European domination over the tropics.

To Her Majesty Marie Henriette, Queen of the Belgians!

The King, your august husband, has deigned to accept the dedication of the book of Palm Trees by my compatriot and friend Mr. Oswald de Kerchove de Denterghem.

When, in my turn, I was called to write THE HISTORY OF ORCHIDS, it seemed to me that I could only publish it under the gracious patronage of Your Majesty.

Palm trees symbolize grandeur, power, majesty; Orchids are grace and elegance; they are Queens in our greenhouses as in the virgin forests of tropical world (Puydt, 1880, p.III).^1^

## Majesties of the plant kingdom

In the nineteenth century, readers attentive to European horticultural publications that discussed the most varied plant families and disseminated practical information about their cultivation, supported by scientific discourse from botany, were accustomed to the position of primacy conferred upon palm trees. “Who is not immediately impressed,” wrote the editor of L’ILLUSTRATION HORTICOLE, in 1855, “by the grand and majestic effect of these plants?” (Quelques..., 1855, p.3). According to the catalog of a horticultural establishment in the city of Porto, taking into consideration the “most beautiful effect,” palm trees occupied “first place,” by “virtue of the elegance of their bearing as well as their magnificent foliage.” Palm trees were believed to uniquely present “a tone of admirable nobility and distinction” (Catálogo..., s.d., p.15).

Although palm trees belong to a diversified family of plants, including some that more closely resemble small shrubs, greater prominence was generally given to genera and species that reached great heights. As was said at the time, this was due to their “lofty bearing,” portraying the idea of sovereignty and elevation, as well as their “beautiful fronds,” associated with the shape of an imperial crown (Araújo, 1854, p.53). The distinction conferred upon the palm tree was also related to its capacity to bear fruit, the longevity of its foliage and also its multiple uses.

In the work THE VEGETABLE KINGDOM, English botanist John Lindley (1866, p.134), founder of the journal THE GARDENERS’ CHRONICLE dedicated to horticulture, emphasized not only the imposing form and the “character of grandeur” of palm trees, but equally their “immense value to mankind,” due to the “numerous objects of economic importance” they could provide. Lindley stressed that, if Alexander von Humboldt, in his journey through Latin America, had mentioned the prodigious capacity of these plants to provide food and beverages such as wine, oil, wax, flour, salt, and sugar, Carl Friedrich Philipp von Martius had also pointed out their importance in providing materials for the manufacture of weapons and dwellings (p.136).

These characteristics were emphasized time after time within a scientistic discourse visibly marked by the imagination of imperial domination. For example, Francisco Duarte d’Almeida e Araújo, the editor of FLORA E POMONA, a journal of agriculture, horticulture, and gardening sponsored by the Portuguese Crown, wrote an article celebrating palm trees in the royal collection. In laudatory terms, he told readers that the “plant kingdom” itself had bestowed upon them the “scepter,” granting them sovereignty over all other plant families. And, he continued: “Their noble and majestic appearance instills respect in those who contemplate them; the trunk, majestic in itself, and their foliage, as gigantic as it is elegant, seem to bear the imprint and mark of the Omnipotent – the seal of Eternity” (Araújo, 1854, p.51).

However, in these discourses, the incomparable grandeur of the palm tree was accompanied by a specific geography: the tropics. British naturalist Alfred Russel Wallace (1853, p.2) emphasized in PALM TREES OF THE AMAZON that “palms are almost exclusively tropical plants,” with few species found in warmer temperate regions. “While the nearer we approach the equator,” he continued, “the more numerous they become both in species and individuals” (p.2). Wallace, like many other men with significant roles in the field of natural sciences, saw palm trees as one of the most “graceful,” “majestic,” and “picturesque” forms of the plant kingdom (p.4).

Palm trees achieved their full splendor in the tropics, as Oswald de Kerchove de Denterghem emphasized in LES PALMIERS: HISTOIRE ICONOGRAPHIQUE (1878), a work dedicated to Leopold II, the King of Belgium. Denterghem, who sought to combine geographical, botanical, and historical knowledge about palm trees in his work, noted exactly this peculiarity: it was in the flora of the tropics that they exhibited their complete grandeur. Thus, they represented the “incomparable power of a nature full of fertility, exuberance, and richness,” resembling the “rich man in his palace” surrounded by everything that makes him “shine,” or even the figure of a “king” that relegates all others to his shadow (Denterghem, 1878, p.1, 90). Collectively, these texts attributed to palm trees terms and expressions of language that made them sovereigns of the tropical plant world. “What grace they display,” wrote the authors of O CAMPO E O JARDIM (1873), “when from their lofty heights they look down regally upon the plebeians, here represented by the humble plants that cluster timidly and grow at their feet!” (Oliveira Júnior, Reis, 1873, p.8).

According to many of the authors that wrote works dedicated to palm trees or collaborated in horticultural journals aimed at a wide audience, this family of plants represented “grandeur, power and majesty” (Puydt, 1880, p.III). These characteristics were derived from a linguistic repertoire frequently used in discourses that referred to the European empires of the period. This was not only due to their height and crown-like foliage, but above all to the presence of an imperial imagination of domination, which, particularly over the course of the nineteenth century, defined the tropics as spaces primarily governed by their plant nature, allowing them to be exploited, appropriated, and colonized by the so-called Old World. While Europe saw its scientific discoveries and its industrial and urban development as indispensable signs of civilization, there was consolidated, in counterpoint, a perception of the tropics as backward, primitive regions dominated by exuberant vegetation. From this perspective, tropical nature was seen as an immense plant kingdom, with palm trees as its majesty.

In dialogue with tropicality studies, which analyze how the tropics were represented in European discourse, this article contributes to the debate by examining European imagination surrounding palm trees. These plants were viewed as figures of sovereignty in territories believed to be dominated by nature and inhabited by peoples considered primitive. As such, I will use a diverse repertoire of texts and images, largely found in ornamental horticulture publications produced in countries such as England, France, Belgium, constantly revered for their conquests and activities, and Portugal, due to its linguistic and cultural proximity to Brazil.

Horticulture gained enormous relevance throughout the nineteenth century, attracting not only natural science readers but also a broader audience of bourgeoisie, nobility, and royalty interested in cultivating species deemed exotic for their tropical origins. With the advance of colonial relations in different parts of the tropical world, these plant species were no longer restricted to botanical gardens. Amid a lucrative plant trade, put into practice by English, Belgian, and French horticulturists, the most diverse specimens also came to figure in the private collections of members of the wealthy classes, who did not hesitate to spend large sums of money on the enhancement of their gardens and greenhouses (El Far, 2025).

It was also in these publications that the counterpoint between civilization, as something belonging to Europe, and backwardness, as a central characteristic of tropical regions, was expressed in a perceptible way. While plants typical of the tropics were exoticized and described against a backdrop of virgin forests and exuberant landscapes, emphasis was placed on the European capacity to know and domesticate, on temperate soil, even those plant families that depended on hot and humid climates to survive.

As shown below, the voluminous financial expenditure directed toward the construction of immense hothouses, capable of housing palm trees of different genera and species from the tropics, was, in my view, entirely in tune with the European imperial sentiment of dominion over these regions. After all, inside these glass palaces, it was possible to admire those that were considered the majesties of that immense, distant, and exotic plant kingdom.

## Palm trees and tropicality

In his analysis of the notion of Orient as a European discursive invention, Edward Said understood representations not as more or less faithful descriptions of reality, but essentially as cultural and political elaborations. The Orient, as recounted in English and French narratives from the late eighteenth century onward, was described as an exotic, backward, sensual, and barbarous place in consonance with a sovereign European consciousness, marked by the desire for colonial domination and exploitation. In this way, a vision of the Orient was delineated that helped “to define Europe (or the West),” in Said’s words, “as its contrasting image, idea, personality, experience” (Said, 1996, p.13-14).^2^

As historian David Arnold adds, parallel to orientalism, there was the discourse of tropicality, thus showing that “historically Europe possessed more than one sense of ‘otherness’” (Arnold, 2000, p.7). According to Arnold, although ideas surrounding the tropics were “deeply ambivalent,” at times emphasizing a place of natural abundance and great fertility, at others exposing a landscape undermined by diseases, plagues, and poverty, as a rule, a common perception was shared: while Europe had achieved “an unprecedented mastery over nature, in the tropics nature’s rule still seemed absolute” (p.11). The reverberation of this vision, which to a great extent constituted the ideological framework that fostered colonial practice, was present not only in the pens of travelers, writers, and visual artists, but figured, in its own way, in technical and scientific works related to disciplines such as botany, geography, anthropology, and zoology (p.7).

That is, the tropics, beyond a cartographic space, denoted a “powerful array of associations” (Driver, 2004, p.2). In a broad and diverse collection of texts and images, one recurrently saw virgin forests and exuberant landscapes, circumscribed by hot climate and by inhabitants morally judged as indolent and lazy, whose knowledge was devalued, thus configuring a world distinct from Europe. The tropics, therefore, were conceived as the opposite of temperate nature, understood in turn as the stage for everything that was considered “modest, civilised, cultivated” (p.3). As historian Nancy Stepan (2001, p.17) affirms, the entrenchment of this “radical otherness” contributed “to the formation of European identity,” which positioned itself as “distinct from that of the tropical zone.”

In their task of analyzing European projections about the tropics, tropicality studies have also pointed to the heterogeneous character of these discourses, emphasizing, as geographer Federico Ferretti (2017, p. 346) stated, the presence, “complexity and the richness of unorthodox voices,” coming from plural individual trajectories. This is because, though produced amid “highly asymmetrical relations of domination and subordination,” as anthropologist Mary Pratt (1999, p.27) points out, many of these narratives emerged within “contact zones,” which she describes as, “social spaces where disparate cultures meet, clash, and grapple with each other.”^3^ It is in this sense, therefore, that historian Felix Driver (2004, p.3-4) proposes the word “transactions” in place of “projections” in an attempt to avoid emphasis on an autonomous and independent symbolic production that imposes itself on the rest of the world, in order to instead recognize the production of knowledge as “a living space of encounter and exchange,” thus including the agency of the represented world.

Amid these reflections, one can take the palm tree as a privileged object for thinking about a certain European perspective that, over the course of the nineteenth century, even while plural and permeated by its particularism, read tropical nature through a common spectrum under the lens of imperialism. Considering a broader historical perspective, it becomes clear that the “complex world” of meanings attributed to the palm tree was not a novelty of the 1800s. According to Spanish philologist José Manuel Díaz de Bustamante (1980, p.28), “the Renaissance and Humanism drew on the best of classical and biblical traditions, deeply rooted in the Middle Ages, and made the palm tree symbol an almost inexhaustible source of new symbols, allegories, and emblems.” Thus, as a polysemic symbol, the palm tree has been present in different political, literary, and religious registers throughout history, on some occasions, for example, signifying victory, as the image of the “triumphal palm” alludes to, on others, eternal life, and in still other contexts, matrimony, due to its sexual reproduction and its capacity to bear fruit (Xavier, Cardim, Álvarez, 1996, p.64).

However, in the nineteenth century specifically, as Nancy Stepan (2001, p.19) points out, palm trees were chosen by Humboldt as “the most noble of tropical plants,” which led to their mere presence evoking, almost “instantly,” “less a botanical species than an imaginative submersion in hot places.” Alexander von Humboldt, accompanied by French botanist Aimé Bonpland, between 1799 and 1804, traveled through various Spanish colonies, including Venezuela, Colombia, Ecuador, Cuba, Mexico, and even passed through the southern United States. Upon his return, he devoted himself to a monumental authorial and editorial work containing more than thirty volumes with technical and detailed descriptions of his journey. First printed in France, it received the title VOYAGE AUX RÉGIONS ÉQUINOXIALES DU NOUVEAU CONTINENT FAIT EN 1799, 1800, 1801, 1802, 1803 ET 1804. A few years later, he would summarize his experience in the tropics in NATURGEMÄLDE (1808), translated the same year into French as TABLEAU DE LA NATURE, in which he brought to the general public his production of knowledge that wove together science, sentiment, and art.

Inspired by German Romanticism, particularly by the thought of Johann Wolfgang von Goethe, of whom he was a close friend, Humboldt believed in the relevance of subjectivity and sensitivity in the observation of nature as well as in the central role of the aesthetic dimension in scientific narrative (Wulf, 2022, p.46). As he himself once affirmed, taking Camões’s work as an example, “rhetorical ornament” was never an “obstacle to the exactitude placed in the presentation of the physical world.” On the contrary, it reinforced the “vividness of impressions left by the grandeur and veracity of nature’s images” (Humboldt, 2007, p.186). The experience of travel thus became indispensable. It was necessary “not only to see with one’s own eyes,” but also, as historian Lorelai Kury (2001, p.879) emphasizes, “to hear and feel with one’s own body the phenomena there where they happen.” By thus laying the foundations of a new discursive terrain about the tropics, Humboldt traced, in Mary Pratt’s words (1999, p.195-196), the general lines of an ideological reinvention of South America “on both sides of the Atlantic.”

Palm trees held a distinctive position in Humboldt’s celebratory narrative of American nature. In VIEWS OF NATURE (1808), specifically in the chapter dedicated to this plant family, Humboldt recounted that, until the death of Carl Linnaeus, in 1778, only fifteen species had been systematically described. He and Bonpland, in his words, had “described 20 new palm species” and “differentiated as many more,” which they named even without having found “complete blooms from them” (Humboldt, 1950, p.59).^4^ The challenge in understanding the physiognomy of palm trees rested, according to him, on three aspects: the short flowering period, the distinct locations of different species across a vast tropical territory, and the difficult access to their inflorescence, situated high up near the foliage, placing the “European” at the mercy of the goodwill of indigenous peoples, whom he described as people immersed in “such intractable coolness.” “People in the tropics,” said Humboldt, “do not take on any strenuous work unless forced to it by the most extreme need” (p.63-64).

For Humboldt, what conferred a majestic aspect upon palm trees was the shape of their foliage, as he himself wrote, “the orientation of its leaves” and the “axis itself.” “The more acute the angle that the fronds create with the (upward) growth of its trunk,” he continued, “the more grand and dignified is its form” (Humboldt, 1950, p.66). Thus, imbued with this perspective, that Humboldt exclaimed “what name shall we give to these majestic plants?” (Humboldt, Bonpland, 1827, p.138).

Alexander von Humboldt’s work on South America became a reference and inspiration for various travelers on their scientific expeditions. Among those who visited Brazil, perhaps Carl Friedrich Phillipp von Martius, as Lorelai Kury (2001, p.866) underscores, “was the most important Humboldtian.” Based on the journey he undertook through Brazilian territory alongside zoologist Johann Baptist von Spix, between 1817 and 1820, Martius published the book TRAVELS IN BRAZIL, composed in its first edition of four volumes, released between 1823 and 1831. Given Spix’s premature death in 1826, in Munich, a large part of the writing fell to Martius, who, like Humboldt, valued the observer’s subjective perception, praising the Brazilian tropical landscape through an “aesthetic-scientific style” (Lisboa, 1997, p.92). One morning in Pará, they wrote that under the “deep blue sky,” one could hear the “whisper of palm trees,” which accompanied the “hosanna intoned by the song of flocks of birds,” in a “superb festival of nature” (Martius, Spix, 2017, p.414).

Martius’s “particular predilection” for palm trees, “these majestic daughters of our planet,” as he himself said, was already expressed in TRAVELS IN BRAZIL, but it would be in his new scientific, editorial, and aesthetic undertaking that the botanist would devote himself to producing an extensive and in-depth study dedicated solely to this plant family (Martius, Spix, 2017, p.194). The publication of HISTORIA NATURALIS PALMARUM, begun in 1823 and completed in 1850, composed of three volumes totaling 550 pages, covered a still unprecedented space in the field of natural sciences and, as biologist Jürke Grau (2012, p.113) affirmed, counted on the collaboration of renowned botanists, showing Martius’s capacity to “bring together scientists of diverse orientations for the benefit of a task.” Taking Brazilian palm trees as his starting point, Martius linked “Brazilian vegetation with the universe of the tropics in a global way,” thereby securing his lasting influence on subsequent botanical studies through the work’s exceptional quality (p.112).

In his 1853 work PALM TREES OF THE AMAZON, Alfred Russel Wallace (1853, p.V-VI) recorded his gratitude for Martius’s “magnificent work,” whose parts on the “determination of the genera and species,” the description of “botanical characters,” and the “geographical distribution of Palms” had been, for him, of special importance. Martius was also exalted by Berthold Seemann in his POPULAR HISTORY OF THE PALMS AND THEIR ALLIES, in 1856, and years later, among others, by Oswald de Kerchove de Denterghem in his LES PALMIERS: HISTOIRE ICONOGRAPHIQUE, from 1878. Beyond his botanical knowledge, Martius was distinguished by the numerous images present in his work, some of them made by himself, which led John Lindley (1866, p.134-136), in THE VEGETABLE KINGDOM, to say that he was “the great illustrator of this noble family.”

**Figure 1:**
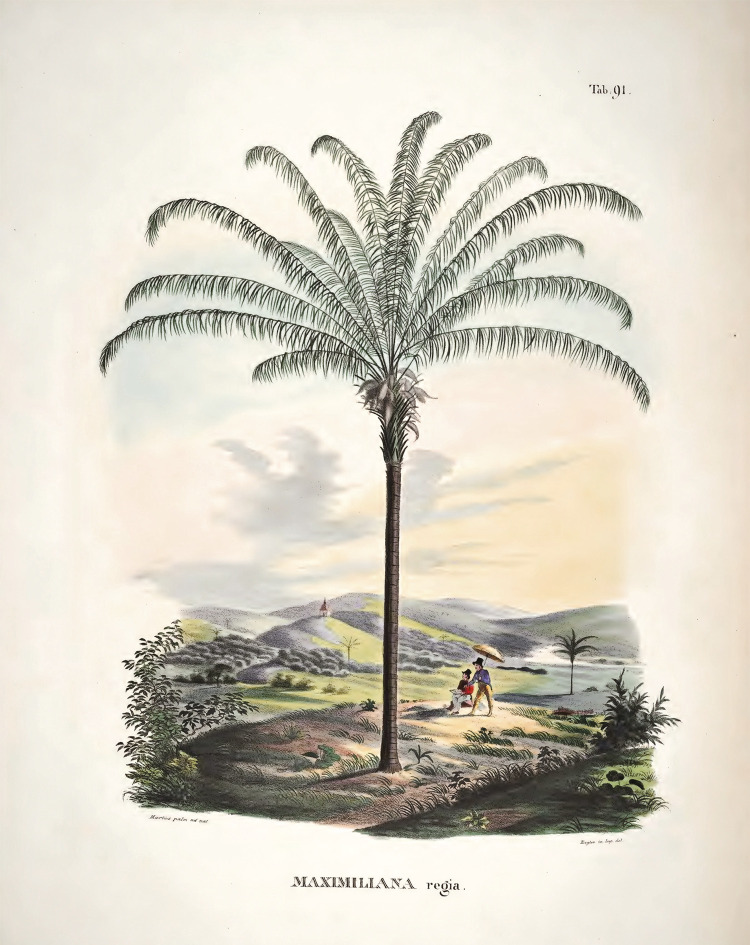
: MAXIMILIANA REGIA, Tab. 91 (Martius, s.d., v. 2).

In one of these illustrations, Martius himself appears depicting a palm tree named MAXIMILIANA REGIA,^5^ a visible tribute to Maximilian I, who had sponsored his Brazilian expedition (Figure 1).

In this illustration, the MAXIMILIANA REGIA is seen in the foreground right at the center of the scene. Some aspects of the palm tree probably dialogue with styles and references coming from the field of botany, such as, for example, the acute angle of most fronds, something that can also be seen in the drawings present in the works of Alfred Russel Wallace and Berthold Seemann. Others, however, can be read considering an aesthetic dimension related both to Humboldt’s work and to landscape painting itself. According to art historian Claudia Valladão de Mattos (2004, p.158), Goethe, and later Humboldt, had been fascinated by the mastery of German painter Jakob Philipp Hackert in “capturing the details of nature, the types of trees, the geography of the landscape and the atmosphere specific to the portrayed location,” in order to then “synthesize these dispersed elements and present them in an essential gaze.” Beyond the details, what mattered in this approach was the creation of a “synthesis-image” capable of producing an effect on the observer. This characteristic would have been adopted by Humboldt and, through his work, it influenced artists who portrayed Brazil, such as Johann Moritz Rugendas, Thomas Ender, and Carl Friedrich Philipp von Martius (Mattos, 2004, p.163).

However, alongside these characteristics, the image produced by Martius holds special interest to this article. In it, Martius and Spix attentively observe the MAXIMILIANA REGIA and appear to converse about what is perceived. In this exercise, no autochthonous person is portrayed. Martius and Spix, elegantly dressed amid the tropical landscape, describe and portray the MAXIMILIANA REGIA, thereby proclaiming the presence of a legitimate and exclusive knowledge that belonged to them.

**Figure 2:**
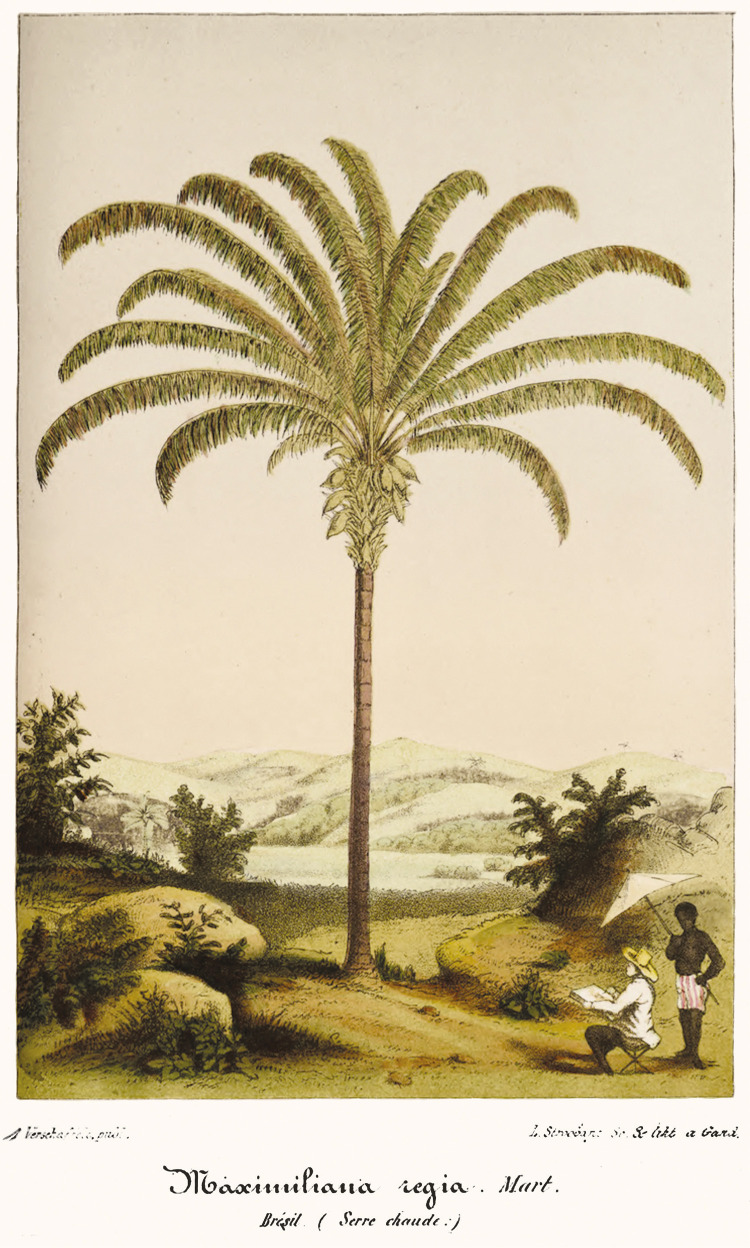
: MAXIMILIANA REGIA Mart. (Quelques..., 1855).

By showing Martius in his fieldwork alongside palm trees, this image probably achieved significant repercussion among the public interested in botanical and horticultural studies. Perhaps for this reason it was published again in 1855 in the respected Belgian horticultural journal L’ILLUSTRATION HORTICOLE, in a section dedicated solely to the MAXIMILIANA REGIA. After paying homage to Martius’s “immortal work” and exalting palm trees for their “supreme beauty” and “extreme usefulness” (Quelques..., 1855, p.1-2), the journal presented a reworking of the image originally published in HISTORIA NATURALIS PALMARUM (Figure 2).

In the new illustration, the MAXIMILIANA REGIA remained at the center of the scene, amid a natural landscape that, though different, retained similarities to the image published in the German botanist’s study. What changed significantly was the figure of Martius himself, now in the foreground, no longer accompanied by Spix, but rather by a Black man, who is there solely to protect him from the sun. In this image published by L’ILLUSTRATION HORTICOLE, the feeling of European sovereignty also appears in the racial question. While the Black man stands behind the scientist with his chest bare, positioned at a distance, Martius conducts his scientific work, properly clothed for fieldwork. If in the image present in HISTORIA NATURALIS PALMARUM, Spix is portrayed in dialogue with Martius, conveying a situation of proximity and knowledge exchange, in the second, the separation and silence are complete.

The image, published in L’ILLUSTRATION HORTICOLE, reinforces the common European perception of tropical populations as removed from civilization, contrasting them with the scientific and technological progress embodied by the European traveler. While the tropical region was distinguished by its exuberant nature, in which the palm tree gained centrality, its population was described as indolent and backward, being incapable of taking advantage of what nature had spontaneously offered them. “Where one could work miracles,” said Julio Rossignon (1866, p.VII-VIII) in his MANUAL DO JARDINEIRO E DO ARBORICULTOR, there was a people deprived of these “artistic views,” for him, due to the “lack of necessary knowledge,” “also because of some apathy of its inhabitants.”

Amid this perspective, horticulture was transformed into an indicator of civilization. In the ALMANACH DO HORTICULTOR, published in the city of Porto, its editor, Oliveira Júnior (1871, p.58), said:

Can one determine a country’s level of civilization by the state of its floriculture? Certainly! Consider France, Belgium, and England, where a genuine passion for plants exists. Are these not the most civilized countries? And as the other side of the coin, we present Africa and America, where despite the favorable climate, no importance whatsoever is given to the cultivation of plants. This is hardly surprising; these are countries still largely deprived of civilization’s light.

With a gaze that did not see the entire colonial project in tropical regions, which for centuries imposed agricultural monoculture, large estates, and slave labor, Oliveira Júnior used ornamental horticulture to reinforce the division between two worlds: the tropics and Europe. The first, for him, dominated by the state of nature, and the second celebrated for the technological advances that allowed the domination of nature.

## Palm houses and the royal palm collections

Since the sixteenth century, European royal and botanical gardens played crucial roles in acclimatizing, cultivating, studying, and exhibiting plant species from colonial territories (Domingues, 2001; Kury, 2004; Sanjad, 2006). To protect these plants from the harsh European winter, hothouses were built with engineering that sought to artificially reproduce the climate of tropical regions. In this context, orangeries gained relevance, coming to feature in nobility properties. It was said that, during his reign, Louis XIV of France had commissioned the construction of two hothouses dedicated to the cultivation of oranges, one of them built at Versailles, in 1685 (Hix, 1996, p.15). With technological advances, glasshouses gradually emerged with their glass walls sustained by a gigantic iron structure, receiving not only heat and humidity but also light throughout the day.

The nineteenth century witnessed the apex of this process, not only due to the architectural development of these buildings, but above all because of the advance of colonial domains intrinsically related to the vertiginous growth of botany and horticulture. With this, public and private properties enhanced their greenhouses and their herbariums with thousands of species from overseas. In the words of a renowned architect of the time, John Claudius Loudon, in his book REMARKS ON THE CONSTRUCTION OF HOTHOUSES, from 1817, these artificially heated greenhouses made it possible “to exhibit spring and summer in the midst of winter, and to bring to perfection the delicious fruits and splendid flowers of the torrid zone in a temperate or cold country,” which gave man “so proud” of himself the “command over Nature” (cited in Kohlmaier, Sartory, 1986, p.26).

Although this feeling of dominion, interwoven with the growth of cities and industrial dynamics, brought to light new values and sensibilities that began to question man’s relationship with nature, this period witnessed a widespread desire for species considered ornamental and exotic, leading numerous collectors to the tropics who did not hesitate to deforest Brazilian forests in the hope of highly lucrative commercial transactions (Thomas, 2010; Dean, 1992, p.13; Stols, 1971).

It was in this scenario that the so-called palm houses gained the attention of European nobility and became privileged symbols of wealth and power. Their elevated height ensured shelter for different genera of palm trees as well as other large plants from hot climates, such as, for example, banana trees. In “nearly every civilized part of Europe … palm houses are built,” wrote naturalist Berthold Seemann (1856, p.37).

According to news published in the English newspaper THE GARDENERS’ CHRONICLE, Friedrich Wilhelm III, then king of Prussia, opened to the public in 1841, in Pfaueninsel, near Potsdam, today Germany, his palm house, one of the most beautiful buildings of this type, thirty meters long and 12 meters high, richly decorated in its interior with artifacts from the Orient, as shown in two oil paintings made by Carl Blechen under royal patronage, both titled DAS INNERE DES PALMENHAUSES (1834). It was reportedly the king himself who bought from the Frenchman M. Fulcheron a large part of his collection formed, among other plant families, by about eighty palm trees of 41 different species, some of them considered genuine treasures. The article said:

Particularly distinguished is a specimen of CHAMAEROPS HUMILIS, which was formerly in the botanic garden at Bonn, and, was brought from thence in 1831. This tree is 300 years old, has a trunk ¼ foot in diameter, and 10 feet in height to the crown, which is 9 feet in diameter. The height of the whole is 16 feet. It blows in the winter months, and bears male flowers, with which the female flowers of the smaller plants were fertilized in February of the year 1834, so that they have already a show of male fruit. LATANIA BORBONICA is remarkable for its fine growth and the richness of its leaves, forty of which form the crown, which is upwards of 24 feet in diameter. ZAMIA TRIDENTATA and REVOLUTA have trunks of 1feet in diameter. A specimen of the Sago Palm, CYCA REVOLUTA, has a trunk 3 feet in height. Several of the plants have flowered (The king..., 1843, p.69).

In the narrative presented, it can be noted that certain qualities were emphasized and listed based on different levels of importance. First came the height reached by each specimen, according to the specificities of the species, and the diameter of its crown. The greater the extension of the trunk, in height and diameter, and the more opulent the foliage of its crown, the more distinguished the palm tree became in the eyes of its admirers. Next, mention was made of its capacity for flowering and fruit production, and, in particular cases, its place of origin and age. Together, these elements seemed to trace a direct line to notions of wealth, power, and vigor of the very “chiefs of states,” to whom palm trees were also often compared, as Berthold Seemann emphasized in his book POPULAR HISTORY OF THE PALMS AND THEIR ALLIES (Seemann, 1856, p.11).

When the Royal Botanic Gardens Kew ceased to be solely a royal garden, in 1840, and became one of the greatest receptacles of plant species from an ever-increasing number of regions annexed to the British Empire, the construction of a palm house was deemed indispensable, whose size and “noble structure” should be unprecedented in Europe. The project, entrusted to Decimus Burton, was completed in 1848 and presented a “novel system of arching iron beams” (Drayton, 2000, p.185) in a building 110 meters long, thirty meters wide, and twenty meters high, whose central area also featured a kind of mezzanine at a height of nine meters from the floor, reached by spiral staircases, thus enabling visitors to see the crowns of the palm trees, even the tallest ones (Seemann, 1856, p.38-39).

**Figure 3:**
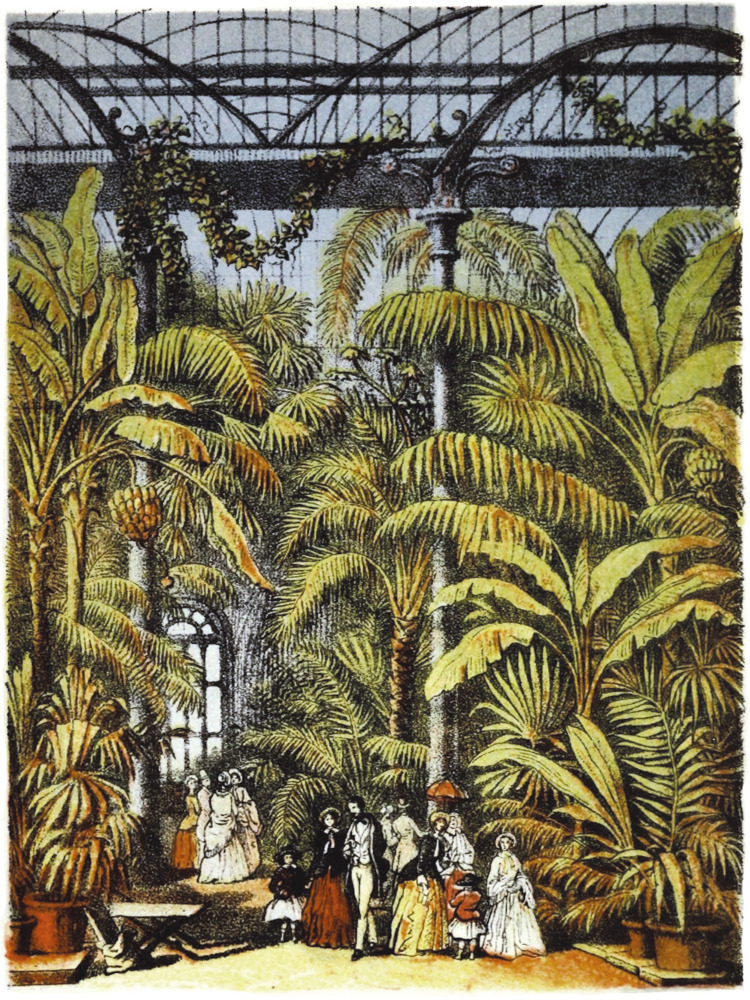
: The great palm house at the Royal Gardens, Kew (Seemann, 1856): Conflict of interests

The interior of the Kew Gardens palm house was displayed by Berthold Seemann in his book about palm trees through an image he named “The great palm house at the Royal Gardens, Kew.” In the illustration, it is possible to see the enormous dimension of the building with glass walls and ceilings, supported by structures and columns made of iron. One also notes profuse vegetation, admired by elegantly dressed couples, children, and ladies (Figure 3).

Such fascination was also highlighted by Seemann in the pages of his book. According to him, the visitor experienced the sensation of “suddenly” finding himself in the midst of dense tropical vegetation composed of banana trees, bamboos, ferns, strelitzias, cacti, vanilla plants, tamarinds, and a “notable” collection of palm trees, which he described thus:

The two loftiest Palms arresting the attention are species of Cocoa-nut (COCOS PLUMOSA and C. CORONATE), both good examples of the extensive group bearing pinnatisect leaves; the two stoutest, a species of Sabal (S. UMBRACULIFERA), equally good examples of another less numerous groups, distinguished by its fan-shaped leaves. There are besides in this collection: – the Date Palm (PHOENIX DACTYLIFERA), producing the dates of commerce and scripture; the Palmyra Palm (BORASSUS FLABELLIFORMIS), one of the most difficult of Palms to rear; the African Oil-palm (ELÆIS GUINEENSIS), which yield Palm-oil; the Cocoa-nut (COCOS NUCIFERA), the uses of which are said to be more numerous than the days of the year; the American Cabbage Palm (OREODOXA OLERACCA), the young leaves of which are an excellent esculent vegetable; the Betel-nut tree (ARECA CATECHU); the Wild Date of India (PHŒNIX SYLVESTRIS), supplying Palm-wine and sugar; the Ivory-plant (PHYTELEPHAS MACROCARPA), the seeds of which resemble animal ivory in appearance; the Wax Palm of the Andes (CEROXYLON ANDICOLA), of which the full-grown stem is covered with a waxy substance; and lastly the Broom Palm (ATTALEA FUNIFERA), the coarse fiber of which is used for making brooms and brushes (Seemann, 1856, p.40-41).^6^

In his account, Seemann allows us to know which palm species composed the Kew Gardens collection in 1856, alluding – as author of a book dedicated to the study and description of palm trees – to their uses, their morphology, and the provenance of each species. Other narratives less supported by the scientific gaze also help us, in their own way, to understand the rapture that the palm trees gathered there caused in the spectator. For example, D. Pedro V, the young king of Portugal and the Algarves, who visited the Kew Gardens palm house in 1854 in the company of Prince Albert, expressed unreservedly in his travel account his admiration for the building’s dimensions, noting that not even a “COCOS PLUMOSA almost perfectly developed” (D. Pedro V, 1923, p.150) could reach the ceiling. Regarding the collection itself, D. Pedro V invited the reader on an imaginary stroll and displayed his marvel at the species collected from different parts of the world. He states:

How shall I describe the beautiful collection of palm trees!? It’s almost impossible. Imagine yourself in a vast grove of LATANIAS, CORYPHAS, SABAL, CHAMAEROPS, CYNOSUREAE, ENCEPHALARTOS, forming the most picturesque groups and dominated by COCOS, by CARYOTAS ... The pen is insufficient to portray the beauties of nature. The impression caused by the bird’s-eye view from the greenhouse balconies is even more impossible to describe. One hovers in thought over Asia, Africa, America, and Oceania and also thinks of Europe, because only there does one see such beautiful things gathered together (D. Pedro V, 1923, p.150).

While large hothouses were indispensable for royal collections located in Northern European countries, in Portugal, by contrast, having the advantage of a milder winter, the open-air growth of a large number of various genera and species of palm trees was witnessed, under the constant care of D. Fernando II – king consort of D. Maria II and father of D. Pedro V – whose interest in gardens and horticulture later made him known as “the landscape king.” With the help of his faithful gardener, Jean Baptiste Bonnard, throughout his entire life D. Fernando II dedicated considerable time and money to the constitution of the garden of the Palace of Pena, the royal summer residence, and the Tapada das Necessidades, located in the Alcântara region of Lisbon. In the words of historian Cristina Castel-Branco (2001, p.87), “D. Fernando chose for Pena a less exuberant vegetation than for Necessidades, in which palm trees are the major novelty.” Thanks to a visit by German botanist Edmond Goeze to the Necessidades garden, recounted in detail in an article published in the JORNAL DE HORTICULTURA PRÁTICA of Porto, in 1876, one can glimpse the genera and species that formed part of D. Fernando II’s royal collection at that time.

Even though the Tapada das Necessidades park was a space that brought together a great variety of bamboos, strelitzias, dracaenas, araucarias, orange trees, lemon trees, fig trees and magnolias, among others, Goeze (1876, p.43) justified beginning his narrative with the palm trees, since they were the “queens of the plant kingdom.” First, the botanist mentioned “the celebrated Chile coco palm (JUBAEA SPECTABILIS),” acquired by D. Fernando 18 years earlier, which had developed there in an “astonishing” manner, at that moment measuring ten meters in height and four meters in circumference, which made it “perhaps the most beautiful of this species in all of Europe” (p.43). Next, Goeze cited the recent acquisition of a LIVISTONA SINENSIS, which was for him a “beautiful Chinese specimen” that competed in beauty with the “robust tree of the species SABAL ADANSONII,” which, in turn, had already borne fruit with good seeds for reproduction. The German botanist also commented on a LIVISTONA AUSTRALIS (CORYPHA AUSTRALIS) and a SEAFORTHIA ELEGANS, both from Australia, whose size and beauty were rarely seen even “in the largest collections of Europe” (p.43).

Goeze (1876, p.43) continued his stroll, pointing also to a group of thirty CHAMAEROPS, composed of four different species, “artistically arranged,” thus forming the “NON PLUS ULTRA of perfection.” He equally mentioned a PHOENIX RECLINATA, which was “well represented” there; a PHOENIX DACTYLIFERA, the species which, in his words, was the “most widespread in Portugal,” “where it sometimes reaches a great height;” and also a COCOS AUSTRALIS, which for him was the “most majestic” palm of Necessidades, reaching 12 to 15 meters in height (p.44).

The fascination with palm trees on European soil gave rise to the construction of various other palm houses in countries such as England, Germany, Austria, Denmark, Scotland, and France. Although glasshouses were not necessary for their reception, in Brazil, palm trees also symbolized, among other aspects, nobility and power. When D. João VI, then prince regent, arrived in Brazil, he personally planted a ROYSTONEA OLERACEA from the Antilles, in 1809, at the newly inaugurated Botanical Garden of Rio de Janeiro. This palm became known as the imperial palm. Over time, this type of palm began to be seen also in public squares, in the gardens of renowned institutions, as well as in the residences of wealthy families, displaying, as was believed, airs of distinction and nobility (D’Elboux, 2006, p.199).

## Final considerations

Humboldt’s early nineteenth-century writings sparked widespread European fascination with palm trees. They were celebrated for their “majestic” form along with their multiple uses by various men of science, particularly from botany and horticulture. Despite these authors’ different trajectories and intellectual perspectives, a common view emerges emphasizing the position of primacy of this plant family in the vast tropical plant kingdom – something that significantly contributed to entrenching an identity experience of contrast. On one side, a region portrayed primarily through its exuberant nature, whose inhabitants were viewed in most accounts as indolent and savage; on the other side, Europe, with its temperate climate, as a model of progress and civilization.

One can say, therefore, that parallel to the emergence of a discursive ensemble that invented the Orient as an exotic, mythical, and fantastical space (Said, 1996), narratives were also produced that defined the tropics through their nature described as exuberant, superb, and spectacular. Tropicality studies have therefore emphasized how this discursive theme sealed in the European imagination the notion of a region dominated by its nature. In this diverse collection of texts and images, which also included horticulture and botanical publications, palm trees received special attention, being described through images and linguistic resources central to the European imperial imagination of the time.

Palm houses can thus be seen as privileged symbols of power and sovereignty. This is because their gigantic and modern architecture displayed wealth and progress while simultaneously demonstrating European dominion over the tropics. After all, under their roofs stood not only a variety of other plants but also palm trees, seen as the very majesty of that distant, fanciful, and immense plant kingdom.

## Data Availability

Not deposited in a data repository.

## NOTES

1 [Translator’s note] In this and other citations of texts from non-English languages, a free translation has been provided.

2 [Translator’s note] Citations in English from Said (1996) were sourced from Edward Said, ORIENTALISM, New York, Vintage House, 1979, p.2.

3 [Translator’s note] Citations in English from Pratt (1999) were sourced from Mary Pratt, IMPERIAL EYES: TRAVEL WRITING AND TRANSCULTURATION, London and New York, Routledge, 2003, p.4.

4 [Translator’s note] Citations in English from Humboldt (1950) were sourced from Alexander von Humboldt, VIEWS OF NATURE, Chicago, The University of Chicago Press, 2014, p.207-210.

5 MAXIMILIANA REGIA, popularly known as INAJÁ, has also received other scientific names, such as ATTALEA MARIPA (Aubl.) Mart. Lorenzi (2010, p.87).

6 In this citation, the popular names of the palm trees mentioned by the author in the English version have been maintained.
