# Supplementary material for: Majestic palm trees: tropical nature and imagination in the context of nineteenth century European imperialism
Source: Hist Cienc Saude Manguinhos. 2025 Sep 22;32:e2025042. doi: 10.1590/S0104-59702025000100042en (PMC12459758; doi:10.1590/S0104-59702025000100042en)
Supplement: Translation

# Translation: Majestosas palmeiras: natureza tropical e imaginário no contexto do imperialismo europeu do século XIX

**DOI:** 10.1590/S0104-59702025000100042

**Authors:** Alessandra El Far

**Affiliations:** i Professora associada, Departamento de Ciências Sociais/Universidade Federal de São Paulo. Guarulhos – SP – Brasil. el.far@unifesp.br

**Keywords:** Palmeira, Tropicalidade, Representação, Imperialismo, Século XIX

## Abstract

Este artigo analisa a maneira pela qual as palmeiras foram representadas, no século XIX, como majestades da natureza tropical por botânicos e horticultores europeus no interior de um imaginário de dominação colonial. Para isso, recupero textos e imagens nas publicações de horticultura ornamental e botânica de países como Inglaterra, França, Bélgica e Portugal, que repetidas vezes veicularam a ideia dos trópicos como um lugar regido por sua natureza exuberante, no qual a palmeira ganhava centralidade, em contraponto à Europa, tomada como modelo de civilização. Era nesse contexto que suntuosas palm houses passaram a ser construídas, atuando também como símbolos privilegiados do domínio europeu sobre os trópicos.

À sua Majestade Marie Henriette, rainha dos belgas!

O Rei, seu augusto esposo, dignou-se a aceitar a dedicatória do livro das Palmeiras de meu compatriota e amigo sr. Oswald de Kerchove de Denterghem.

Quando, por minha vez, fui chamado a escrever A história das orquídeas, pareceu-me que só poderia publicá-la sob o gentil patrocínio de Vossa Majestade.

As palmeiras simbolizam a grandeza, o poder, a majestade, as orquídeas são a graça e a elegância; elas são rainhas em nossas estufas como nas florestas virgens do mundo tropical (Puydt, 1880, p.III).

## Majestades do reino vegetal

No século XIX, os leitores atentos às publicações europeias de horticultura que discorriam sobre as mais variadas famílias de plantas e divulgavam informações práticas sobre o seu cultivo, amparadas por um discurso científico vindo da botânica, estavam acostumados ao lugar de primazia conferido às palmeiras. “Quem não fica imediatamente impressionado”, escrevia o redator de L’Illustration Horticole, em 1855, “com o efeito grandioso e majestoso destas plantas” (Quelques..., 1855, p.3). De acordo com o catálogo de um estabelecimento de horticultura da cidade do Porto, levando em consideração o “mais belo efeito”, as palmeiras ocupavam “o primeiro lugar”, em “virtude da elegância do seu porte como pela sua magnífica folhagem”. Acreditava-se que, de modo singular, as palmeiras apresentavam “um tom de nobreza e distinção admiráveis” (Catálogo..., s.d., p.15).

Embora as palmeiras sejam de uma família diversificada de plantas, incluindo tipos que mais se assemelham a pequenos arbustos, eram os gêneros e as espécies que alcançavam grande altura que geralmente recebiam destaque. Isso se devia, como se dizia na época, ao seu “porte altivo”, vinculado à ideia de soberania e elevação, e às suas “belas frondes”, associadas ao formato de uma coroa imperial (Araújo, 1854, p.53). A distinção conferida à palmeira também estava relacionada à sua capacidade de dar frutos, à longevidade de sua folhagem e também aos seus múltiplos usos.

Na obra The vegetable kingdom, o botânico inglês John Lindley (1866, p.134), fundador da revista The Gardeners’ Chronicle, dedicada à horticultura, salientou não apenas a forma imponente e o “caráter de grandeza” das palmeiras, mas igualmente seu “imenso valor para a humanidade”, em função dos “numerosos objetos de importância econômica” que poderiam fornecer. Lindley ressaltava que, se Alexander von Humboldt, em sua viagem pela América Latina, havia mencionado a prodigiosa capacidade dessas plantas de fornecer alimentos e bebidas como vinho, óleo, cera, farinha, sal e açúcar, Carl Friedrich Philipp von Martius havia apontado também a importância delas na provisão de materiais para a fabricação de armas e habitações (p.136).

Em conjunto, essas características foram ressaltadas vez após vez no interior de um discurso cientificista, visivelmente marcado por um imaginário de dominação imperial. Na publicação Flora e Pomona, por exemplo, um jornal de agricultura, horticultura e jardinagem patrocinado pelos governantes da coroa portuguesa, o redator Francisco Duarte d’Almeida e Araújo, em um artigo que discorria sobre as palmeiras, a fim de valorizar os gêneros presentes na coleção real, dizia aos leitores, em tom laudatório, que o próprio “reino vegetal” havia concedido a elas o “cetro”, conferindo-lhes soberania sobre as demais famílias de plantas. E, continuava: “O seu aspecto, nobre e majestoso, infunde respeito a quem as contempla; e o tronco, que de si já é majestático, e a frondosidade sua, tão gigantesca quanto elegante, parece trazer impresso e marcado, pelo dedo do Onipotente, e o selo da Eternidade” (Araújo, 1854, p.51).

Entretanto, a incomparável imponência da palmeira vinha, nesses discursos, acompanhada de uma geografia específica: os trópicos. Como salientava o naturalista britânico Alfred Russel Wallace (1853, p.2) em sua obra Palm trees of the Amazon, “as palmeiras eram quase exclusivamente plantas tropicais”, com poucas espécies encontradas nas partes mais quentes da zona temperada. “Quanto mais nos aproximamos do equador”, continuava ele, “mais numerosas elas se tornam tanto em espécies quanto em indivíduos” (p.2). Wallace, semelhante a muitos outros homens com relevante atuação no campo das ciências naturais, via as palmeiras como uma das formas mais “graciosas”, “majestosas” e “pitorescas” do reino vegetal (p.4).

Era também nos trópicos, salientava Oswald de Kerchove de Denterghem, autor do livro Les palmiers: histoire iconographique, de 1878, dedicado a Leopoldo II, rei da Bélgica, que elas obtinham todo o seu esplendor. Denterghem, que buscava aliar em sua obra conhecimento geográfico, botânico e histórico sobre as palmeiras, assinalava justamente essa peculiaridade, ou seja, era na flora dos trópicos que elas exibiam sua completa grandeza. Por isso, elas equivaliam, para ele, ao “poder incomparável de uma natureza plena de fertilidade, exuberância e riqueza”, podendo, com isso, assemelhar-se ao “rico em seu palácio”, cercado de tudo o que o faz “brilhar”, ou então à figura de um “rei”, que relegava todos os demais à sua sombra (Denterghem, 1878, p.1, 90). Em comum, esses textos atribuíam às palmeiras termos e expressões de linguagem que faziam delas soberanas da natureza vegetal dos trópicos. “Que donaire não ostentam”, escreviam em 1873 os autores do livro O campo e o jardim, “quando lá do alto olham soberanamente de soslaio a plebe que aqui se acha representada pelas humildes plantas que, tímidas, se agrupam e crescem a seus pés!” (Oliveira Júnior, Reis, 1873, p.8).

Na visão de muitos desses autores, que escreviam obras dedicadas às palmeiras ou colaboravam em revistas de horticultura voltadas também a um público amplo, essa família de plantas representava “grandeza, poder e majestade” (Puydt, 1880, p.III). Essas características eram derivadas de um acervo linguístico frequentemente utilizado nos discursos que se referiam aos impérios europeus da época. Isso não se devia apenas à sua altura e ao formato de coroa de sua folhagem, mas, sobretudo, à presença de um imaginário de dominação imperial que, no decorrer do século XIX em particular, definiu os trópicos como espaços eminentemente governados por sua natureza vegetal, o que permitiu que fossem explorados, apropriados e colonizados pelo chamado Velho Mundo. Enquanto a Europa via suas descobertas científicas e o seu desenvolvimento industrial e urbano como sinais imprescindíveis de civilização, consolidava-se, em contraponto, uma percepção dos trópicos como regiões atrasadas, primitivas e dominadas por uma exuberante vegetação. Nessa perspectiva, a natureza tropical era vista como um imenso reino vegetal, sendo as palmeiras sua majestade.

Isto posto, em diálogo com os estudos da tropicalidade, que analisam a representação dos trópicos nos diferentes discursos europeus, este artigo tem o propósito de contribuir com tal debate lançando luz à existência de um imaginário europeu em torno das palmeiras, vistas como figuras de soberania, em um território, acreditava-se, dominado por uma natureza vegetal e habitado por povos em um estágio primitivo de desenvolvimento. Para isso, utilizarei um diversificado repertório de textos e imagens, em grande parte, presentes nas publicações de horticultura ornamental produzidas em países como Inglaterra, França, Bélgica, reverenciados constantemente por suas conquistas e atividades, e Portugal, por sua proximidade linguística e cultural com o Brasil.

É importante destacar que a horticultura ganhou enorme relevância ao longo do século XIX, atraindo a atenção de leitores das ciências da natureza, mas também de um público alargado, composto por membros da burguesia, nobreza e realeza, interessado em conhecer e cultivar espécies consideradas exóticas, por virem dos trópicos. Com o avanço das relações coloniais, em diferentes partes do mundo tropical, essas espécies vegetais não mais ficavam restritas aos jardins botânicos. Em meio a um lucrativo comércio de plantas, colocado em prática por horticultores ingleses, belgas e franceses, os mais diversos exemplares passaram a figurar também nas coleções privadas de membros das camadas abastadas, que não hesitavam em gastar verdadeiras somas de dinheiro na incrementação de seus jardins e estufas (El Far, 2025).

Era também nessas publicações que o contraponto entre civilização, como algo pertencente à Europa, e atraso, como característica central das regiões tropicais, expressava-se de modo perceptível. Ao passo que as plantas típicas dos trópicos eram exotizadas e descritas tendo por cenário florestas virgens e paisagens exuberantes, destacava-se a capacidade europeia de conhecer e domesticar, em solo temperado, até mesmo famílias de plantas que dependiam do clima quente e úmido para sobreviver.

O volumoso dispêndio financeiro direcionado, como se verá a seguir, para a construção de imensas estufas aquecidas, capazes de abrigar palmeiras de diferentes gêneros e espécies vindas dos trópicos, estava, a meu ver, em plena sintonia com o sentimento imperial europeu de domínio sobre essas regiões. Afinal, no interior desses palácios envidraçados seria possível admirar aquelas que eram consideradas as majestades daquele imenso, distante e exótico reino vegetal.

## Palmeiras e tropicalidade

Em sua análise sobre a noção de Oriente como uma invenção discursiva europeia, Edward Said tomou as representações não como descrições mais ou menos fiéis à realidade, mas essencialmente como elaborações culturais e políticas. O Oriente, tal como relatado nas narrativas inglesas e francesas, a partir do final do século XVIII, foi descrito como um lugar exótico, atrasado, sensual e bárbaro, em consonância com uma consciência soberana europeia, marcada pelo desejo de dominação e exploração colonial. Desse modo, delineava-se uma visão acerca do Oriente, que ajudava a “definir a Europa (ou Ocidente)”, nas palavras de Said (1996, p.13-14), “como sua imagem, ideia, personalidade e experiência de contraste”.

Como acrescenta o historiador David Arnold (2000, p.7), paralelamente ao orientalismo, havia o discurso da tropicalidade, mostrando, assim, que “historicamente a Europa possuía mais de um sentido de alteridade”. Segundo Arnold, embora as ideias em torno dos trópicos fossem “profundamente ambivalentes”, ao enfatizarem ora um lugar de abundância natural e grande fertilidade, ora expondo uma paisagem solapada por doenças, pragas e pobreza, via de regra compartilhava-se uma percepção comum: enquanto a Europa havia conquistado “um domínio sem precedentes sobre a natureza, nos trópicos o governo da natureza ainda parecia absoluto” (p.11). A reverberação dessa visão, que, em grande medida, constituiu o arcabouço ideológico que fomentou a prática colonial, estava presente não somente na pena de viajantes, escritores e artistas visuais, mas figurava, a seu modo, nos trabalhos técnicos e científicos relacionados a disciplinas como botânica, geografia, antropologia e zoologia (p.7).

Ou seja, os trópicos, para além de um espaço cartográfico, denotavam um “poderoso conjunto de associações” (Driver, 2004, p.2). Em um amplo e diversificado acervo de textos e imagens, via-se recorrentemente florestas virgens e paisagens exuberantes, circunscritas pelo clima quente e por habitantes moralmente julgados como indolentes e preguiçosos, cujos saberes eram desvalorizados, configurando, assim, um mundo distinto da Europa. Os trópicos, portanto, eram concebidos no sentido oposto da natureza temperada entendida, por sua vez, como palco para tudo aquilo que se considerava “modesto, civilizado, cultivado” (Driver, p.3). Como afirma a historiadora Nancy Stepan (2001, p.17), o enraizamento dessa “alteridade radical” contribuiu “para a formação da identidade europeia”, que se colocou como “distinta daquela do mundo tropical”.

Em sua tarefa de analisar as projeções europeias acerca dos trópicos, estudos da tropicalidade também têm apontado para o caráter heterogêneo desses discursos, salientando, como afirmou o geógrafo Federico Ferretti (2017, p.346), a presença, “complexidade e riqueza de vozes não ortodoxas”, vindas de trajetórias individuais plurais. Isto porque, embora produzidos em meio a “relações extremamente assimétricas de dominação e subordinação”, como aponta a antropóloga Mary Pratt (1999, p.27), muitas dessas narrativas vieram à tona no interior de “zonas de contato”; em suas palavras, “espaços sociais onde culturas díspares se encontram, se chocam, se entrelaçam uma com a outra”. É nesse sentido, portanto, que o historiador Felix Driver (2004, p.3-4) propõe a palavra “transações”, no lugar de “projeções”, na tentativa de evitar a ênfase em uma produção simbólica autônoma e independente, que se impõe ao resto do mundo, para, diferentemente, reconhecer a produção de conhecimento “como espaço vivo de encontro e troca”, incluindo, dessa maneira, a agência do mundo representado.

Em meio a essas reflexões, pode-se tomar a palmeira como objeto privilegiado para se pensar certa perspectiva europeia, no decorrer do século XIX, que mesmo plural e permeada por seus particularismos, lia, em um espectro comum, a natureza tropical sob as lentes do imperialismo. Levando em consideração uma temporalidade mais alargada, logo se vê que o “complexo mundo” de significados atribuídos à palmeira não foi uma novidade dos Oitocentos. De acordo com o filólogo espanhol José Manuel Díaz de Bustamante (1980, p.28), “o Renascimento e o Humanismo aproveitaram o melhor das tradições clássicas e bíblicas, profundamente enraizadas na Idade Média, e fizeram do símbolo da palmeira uma fonte quase inesgotável de novos símbolos, alegorias e emblemas”. Ou seja, como símbolo polissêmico, a palmeira esteve presente em diferentes registros políticos, literários e religiosos no decorrer da história. Em algumas ocasiões, por exemplo, significando a vitória, como alude a imagem da “palma triunfal”; em outras, a vida eterna; e, ainda, em outros contextos, o matrimônio, em função de sua reprodução sexuada e sua capacidade de gerar frutos (Xavier, Cardim, Álvarez, 1996, p.64).

Entretanto, no século XIX especificamente, como aponta Nancy Stepan (2001, p.19), as palmeiras foram eleitas por Humboldt como “as mais nobres das plantas tropicais”, o que propiciou que a sua mera presença passasse a evocar, quase que “instantaneamente”, “menos uma espécie botânica do que uma submersão imaginativa em lugares quentes”. Como se sabe, Alexander von Humboldt, acompanhado do botânico francês Aimé Bonpland, entre 1799 e 1804, percorreu várias das colônias espanholas, como Venezuela, Colômbia, Equador, Cuba, México, chegando a passar também pelo sul dos EUA. Em seu retorno, empenhou-se em um monumental trabalho autoral e editorial, contendo mais de trinta volumes com descrições técnicas e detalhadas de sua jornada, que, primeiramente impresso na França, recebeu o título de Voyage aux régions équinoxiales du Nouveau Continent fait en 1799, 1800, 1801, 1802, 1803 et 1804. Poucos anos depois, sintetizaria sua experiência nos trópicos em Naturgemälde (1808), traduzido no mesmo ano para o francês como Tableau de la Nature, no qual levava para o grande público sua produção de conhecimento que alinhavava ciência, sentimento e arte.

Inspirado pelo romantismo alemão, particularmente, pelo pensamento de Johann Wolfgang von Goethe, de quem era amigo próximo, Humboldt acreditava na relevância da subjetividade e da sensibilidade na observação da natureza como também no papel central da dimensão estética na narrativa científica (Wulf, 2022, p.46). Como ele mesmo afirmou, certa vez, tomando a obra de Camões como exemplo, o “ornamento retórico” nunca foi “obstáculo à exatidão posta na apresentação do mundo físico”. Pelo contrário, reforçava a “vivacidade das impressões deixadas pela grandiosidade e pela veracidade das imagens da natureza” (Humboldt, 2007, p.186). A experiência da viagem, assim, tornava-se imprescindível. Era preciso “não apenas ver com os próprios olhos”, mas também, como salienta a historiadora Lorelai Kury (2001, p.879), “ouvir e sentir com o próprio corpo os fenômenos lá onde acontecem”. Ao lançar, desse modo, as bases de um novo terreno discursivo acerca dos trópicos, Humboldt traçou, nas palavras de Mary Pratt (1999, p.195-196), as linhas gerais de uma reinvenção ideológica da América do Sul “nos dois lados do Atlântico”.

A narrativa de Humboldt, que tanto celebrou a natureza americana, ofereceu um lugar distinto às palmeiras. Em Quadros da natureza (1808), especificamente, no capítulo dedicado a essa família de plantas, Humboldt contava que até a morte de Carlos Lineu, em 1778, apenas 15 espécies haviam sido descritas sistematicamente. Ele e Bonpland, em suas palavras, teriam “descoberto” “vinte novas espécies de palmeiras” e “reconhecido outras vinte”, que eles nomearam mesmo sem terem encontrado “exemplares completos de suas flores” (Humboldt, 1950, p.59). O desafio em conhecer a fisionomia das palmeiras repousava, segundo ele, em três aspectos: no curto período de floração, nas localizações distintas das diferentes espécies em um vasto território tropical e no difícil acesso à sua inflorescência, situada no alto junto à folhagem, colocando o “viajante europeu” à mercê da boa vontade dos indígenas, que ele descrevia como pessoas imersas em uma “apatia intolerável”. “Nos trópicos”, afirmava Humboldt (1950, p.63-64), “o homem não é ativo senão quando obrigado por uma necessidade absoluta”.

Para Humboldt, o que conferia às palmeiras aspecto majestoso era o formato de sua folhagem, como ele mesmo escreveu, “a direção das folhas” e o “suco traçado pelo seu ráquis”. “Quanto mais agudo é o ângulo que as palmas formam com o prolongamento superior do caule”, continuava, “tanto mais nobre e grandioso é o seu aspecto” (Humboldt, 1950, p.66). Era, portanto, imbuído desse olhar, que Humboldt exclamava “que nome poderemos dar a estas majestosas plantas?” (Humboldt, Bonpland, 1827, p.138).

A obra de Alexander von Humboldt sobre a América do Sul tornou-se referência e inspiração para diversos viajantes em suas expedições científicas. Daqueles que visitaram o Brasil, talvez Carl Friedrich Phillipp von Martius, como sublinha Lorelai Kury (2001, p.866), “tenha sido o mais importante humboldtiano”. Da viagem que realizou pelo território brasileiro ao lado do zoólogo Johann Baptist von Spix, entre 1817 e 1820, Martius publicou o livro Viagem pelo Brasil, composto, em sua primeira edição, por quatro volumes, lançados entre 1823 e 1831. Dada a morte prematura de Spix, em 1826, em Munique, coube a Martius grande parte do trabalho de escrita, que, semelhante a Humboldt, valorizou a percepção subjetiva do observador, louvando a paisagem tropical brasileira em meio a um “estilo estético-científico” (Lisboa, 1997, p.92). Certa manhã, no Pará, eles escreveram que sob o “azul profundo do céu” ouvia-se o “sussurro das palmeiras”, que acompanhava o “hosana entoado pelo canto de bandos de pássaros”, em uma “soberba festa da natureza” (Martius, Spix, 2017, p.414).

A “particular predileção” de Martius pelas palmeiras, “essas majestosas filhas do nosso planeta”, como ele mesmo dizia, já estava expressa em Viagem pelo Brasil, mas seria em seu novo empreendimento científico, editorial e estético, que o botânico se empenharia em produzir um estudo extenso e aprofundado unicamente dedicado a essa família de plantas (Martius, Spix, 2017, p.194). A publicação da Historia naturalis palmarum, iniciada em 1823 e finalizada em 1850, composta por três volumes, somando ao todo 550 páginas, cobriu um espaço ainda inédito no campo das ciências naturais e, como afirmou o biólogo Jürke Grau (2012, p.113), contou com a colaboração de renomados botânicos, mostrando a capacidade de Martius em “confluir cientistas de diversas orientações em benefício de uma tarefa”. Ao tomar como ponto de partida as palmeiras do Brasil, Martius vinculou “a vegetação brasileira com o universo dos trópicos de forma global”, selando igualmente, em função de sua reverenciada qualidade, seu nome nos demais estudos que viriam a seguir (p.112).

Em sua obra Palm trees of the Amazon, de 1853, Alfred Russel Wallace (1853, p.V-VI) registrava sua gratidão ao “magnífico trabalho” de Martius, cujas partes sobre a “determinação dos gêneros e espécies”, a descrição dos “caracteres botânicos” e a “distribuição geográfica das palmeiras” teriam sido, para ele, de especial importância. Martius também foi exaltado por Berthold Seemann em sua Popular history of the palms and their allies, de 1856, e anos depois, entre outros, por Oswald de Kerchove de Denterghem, em sua Les palmiers: histoire iconographique, de 1878. Além de seu conhecimento botânico, Martius foi notabilizado pelas inúmeras imagens presentes em sua obra, algumas delas feitas por ele mesmo, o que levou John Lindley (1866, p.134-136), em The vegetable kingdom, a dizer que era ele “o grande ilustrador desta nobre família”.

Em uma dessas ilustrações, o próprio Martius aparece retratando uma palmeira denominada por ele Maximiliana regia,^1^ em uma visível homenagem a Maximiliano I, que havia patrocinado sua viagem científica pelo Brasil (Figura 1).

**Figura 1:** : Maximiliana regia, Tab. 91 (Martius, s.d., v.2).

Nessa ilustração, a Maximiliana regia é vista em primeiro plano bem no centro da cena. Alguns aspectos da palmeira, provavelmente, dialogam com estilos e referências advindos do campo da botânica, como, por exemplo, o ângulo agudo da maioria das palmas, algo que se pode ver também nos desenhos presentes nas obras de Alfred Russel Wallace e Berthold Seemann. Outros, porém, podem ser lidos levando em consideração uma dimensão estética relacionada tanto com a obra de Humboldt quanto com a própria pintura de paisagem. De acordo com a historiadora da arte Claudia Valladão de Mattos (2004, p.158), Goethe e, mais tarde, Humboldt teriam ficado fascinados com a maestria do pintor alemão Jakob Philipp Hackert em “captar os detalhes da natureza, os tipos de árvores, a geografia da paisagem e a atmosfera própria ao local retratado”, para então “efetuar a síntese desses elementos dispersos e apresentá-los em um olhar essencial”. Para além dos detalhes, interessava, nesse viés, a realização de uma “imagem-síntese” capaz de produzir um efeito sobre o observador. Tal característica teria sido adotada por Humboldt e, por meio de sua obra, influenciado artistas que retrataram o Brasil, como Johann Moritz Rugendas, Thomas Ender e Carl Friedrich Philipp von Martius (Mattos, 2004, p.163).

No entanto, paralelamente a essas características, há na imagem produzida por Martius um aspecto de especial interesse para este artigo. Nela, Martius e Spix observam atentamente a Maximiliana regia e parecem conversar sobre o que é percebido. Nesse exercício, nenhuma pessoa autóctone é retratada. São eles, vestidos com trajes elegantes em meio à paisagem tropical, que descrevem e retratam a Maximiliana regia, proclamando, assim, a presença de um saber legítimo e único, que a eles pertencia.

Por mostrar Martius em seu trabalho de campo junto às palmeiras, essa imagem provavelmente alcançou significativa repercussão entre o público interessado nos estudos de botânica e horticultura. Talvez, por isso, teria sido publicada novamente, em 1855, na respeitada revista de horticultura belga L’Illustration Horticole, em uma seção dedicada unicamente à Maximiliana regia. Após render homenagem à “obra imortal” de Martius e exaltar as palmeiras por sua “beleza suprema” e “extrema utilidade” (Quelques..., 1855, p.1-2), a revista apresentava uma reelaboração da imagem originalmente publicada na Historia naturalis palmarum (Figura 2).

**Figura 2:** : Maximiliana regia Mart. (Quelques..., 1855).

Na nova ilustração, a Maximiliana regia permanecia no centro da cena, em meio a uma paisagem natural que, mesmo diferente, conservava semelhanças com a imagem veiculada no estudo do botânico alemão. O que mudava significativamente era a figura do próprio Martius, agora em primeiro plano, não mais acompanhado de Spix, e sim de um homem negro, que está ali unicamente para o proteger do sol. Nessa imagem veiculada pela L’Illustration Horticole, o sentimento de soberania europeia aparece também na questão racial. Enquanto o homem negro está com o peito nu, atrás e um pouco distante do cientista, Martius está realizando sua atividade científica, com roupa de trabalho de campo. Se, na imagem presente em Historia naturalis palmarum, Spix é retratado em diálogo com Martius, transparecendo uma situação de proximidade e troca de conhecimento, na segunda, a separação e o silêncio são completos.

A imagem publicada na L’Illustration Horticole também reifica, à sua maneira, a visão europeia recorrente de uma população dos trópicos composta por indivíduos apartados da civilização, em contraste com o avanço científico e tecnológico corporificado aqui na figura do viajante. Ao passo que a região tropical primava por sua natureza exuberante, na qual a palmeira ganhava centralidade, sua população vinha descrita como indolente e atrasada, sendo incapaz de tirar proveito do que a natureza lhe havia espontaneamente oferecido. “Onde se pode fazer prodígios”, dizia Julio Rossignon (1866, p.VII-VIII), em seu Manual do jardineiro e do arboricultor, estava um povo privado dessas “vistas artísticas”, para ele, em função da “falta de conhecimentos necessários”, “também por alguma apatia dos seus habitantes”.

Em meio a essa perspectiva, a horticultura era transformada em um indicador de civilização. No Almanach do Horticultor, publicado na cidade do Porto, seu redator, Oliveira Júnior (1871, p.58), dizia:

Poderá conhecer-se o estado da civilização de um país pelo estado da floricultura?

Com certeza! Senão vejamos a França, a Bélgica, a Inglaterra, onde há uma verdadeira paixão pelas plantas, se são ou não os países mais civilizados.

E como reverso da medalha apresentamos a África e a América, onde o clima é tão favorável, e onde não se liga importância alguma à cultura das plantas.

Não admira; são países ainda em grande parte carecidos da luz da civilização.

Com um olhar que não enxergava todo o projeto colonial nas regiões tropicais, que por séculos impôs a monocultura agrícola, o latifúndio e o trabalho escravo, Oliveira Júnior tomava a horticultura ornamental para reforçar a divisão entre dois mundos: os trópicos e a Europa. O primeiro, para ele, dominado pelo estado de natureza, e o segundo celebrado pelos avanços tecnológicos que permitiam o domínio da natureza.

## Palm houses e as coleções reais de palmeiras

Os jardins reais e botânicos europeus tiveram, desde o século XVI, um importante papel na aclimatação, no cultivo, no estudo e na exibição das espécies vegetais trazidas dos domínios coloniais (Domingues, 2001; Kury, 2004; Sanjad, 2006). Para proteger essas plantas do rigoroso inverno europeu, as greenhouses foram construídas com uma engenharia que procurava reproduzir artificialmente o clima das regiões tropicais. Nesse contexto, ganharam relevância as orangeries, que passaram a figurar em propriedades da nobreza. Dizia-se que Luís XIV da França, durante seu reinado, havia encomendado a construção de duas estufas voltadas para o cultivo de laranjas, uma delas edificada em Versalhes em 1685 (Hix, 1996, p.15). Pouco a pouco, com os avanços tecnológicos, surgiram as glasshouses, que, por conta das suas paredes de vidro, sustentadas por uma gigantesca estrutura de ferro, recebiam, além do calor e da umidade, luz durante todo o dia.

O século XIX conheceu o ápice desse processo, não apenas em razão do desenvolvimento arquitetônico desses edifícios, mas, sobretudo, pelo avanço dos domínios coloniais intrinsecamente relacionado ao vertiginoso crescimento da botânica e da horticultura. Com isso, propriedades públicas e privadas incrementaram suas estufas e seus herbários com milhares de espécies vindas do além-mar. Nas palavras de um conceituado arquiteto da época, John Claudius Loudon, em seu livro Remarks on the construction of hothouses, de 1817, essas estufas aquecidas artificialmente possibilitavam “exibir a primavera e o verão em pleno inverno, e trazer à perfeição deliciosos frutos e esplêndidas flores das zonas tórridas em um país temperado ou frio”, o que dava ao homem “tão orgulhoso” de si o “comando sobre a natureza” (citado em Kohlmaier, Sartory, 1986, p.26).

Embora esse sentimento de domínio, entrelaçado ao crescimento das cidades e da dinâmica industrial, trouxesse à tona novos valores e sensibilidades que passaram a questionar a relação do homem com a natureza, presenciou-se nesse período um largo desejo por espécies consideradas ornamentais e exóticas, levando inúmeros coletores aos trópicos que não hesitavam em desmatar as florestas brasileiras na esperança de transações comerciais altamente lucrativas (Thomas, 2010; Dean, 1992, p.13; Stols, 1971).

Foi nesse cenário que as chamadas palm houses ganharam a atenção da nobreza europeia e se tornaram símbolos privilegiados de riqueza e poder. Sua elevada altura garantia o abrigo de diferentes gêneros de palmeiras, como também de outras plantas de grande porte vindas de climas quentes, como, por exemplo, as bananeiras. Em “quase todas as partes civilizadas da Europa ... palm houses são construídas”, escreveu o naturalista Berthold Seemann (1856, p.37).

De acordo com uma notícia publicada no jornal inglês The Gardeners’ Chronicle, Friedrich Wilhelm III, então rei da Prússia, abriu ao público, em 1841, em Pfaueninsel, perto de Postdam, hoje Alemanha, sua palm house, “uma das mais bonitas edificações deste tipo”, com trinta metros de extensão e 12 metros de altura, ricamente decorada em seu interior com artefatos vindos do Oriente, como mostram duas pinturas a óleo feitas por Carl Blechen, sob o patrocínio real, ambas intituladas Das Innere des Palmenhauses (1834). Teria sido o próprio rei a comprar do francês M. Fulcheron grande parte da sua coleção formada, em meio a outras famílias de plantas, por cerca de oitenta palmeiras de 41 espécies diferentes, algumas delas consideradas genuínas preciosidades. Dizia o artigo:

Particularmente notável é um exemplar de Chamaerops humilis, que anteriormente estava no Jardim Botânico de Bonn e foi trazido de lá em 1831. Esta árvore tem trezentos anos, um tronco com 7,5 centímetros de diâmetro e três metros de altura até a coroa, que tem quase três metros de diâmetro. A altura do todo é de quase cinco metros. Rebentam nos meses de inverno e apresentam flores masculinas, com as quais as flores femininas das plantas menores foram fecundadas em fevereiro de 1834, já oferecendo uma mostra de frutos masculinos. Latania borbonica se destaca pelo crescimento fino e pela riqueza de suas folhas, quarenta das quais formam a copa, que tem mais de sete metros de diâmetro. Zamia tridentata e revoluta têm troncos de trinta centímetros de diâmetro. Uma espécie da palmeira sagu, Cycas revoluta, tem um tronco de quase um metro de altura. Várias das plantas têm florescido (The king..., 1843, p.69).^2^

Na narrativa apresentada, pode-se notar que algumas qualidades eram enfatizadas e elencadas a partir de diferentes níveis de importância. Primeiramente, vinha a altura alcançada por cada exemplar, de acordo com as especificidades da espécie, e o diâmetro de sua copa. Quanto maior a extensão do tronco, em altura e diâmetro, e mais opulenta fosse a folhagem de sua coroa, mais distinta a palmeira tornava-se aos olhos de seus admiradores. Em seguida, mencionava-se sua capacidade de floração e produção de frutos, e, em casos particulares, o lugar da procedência e sua idade. Em conjunto, esses elementos pareciam traçar uma linha direta com as noções de riqueza, poder e vigor dos próprios “chefes de estado”, a quem as palmeiras também costumavam ser comparadas, como salientou Berthold Seemann em seu livro Popular history of the palms and their allies (Seemann, 1856, p.11).

Quando, em 1840, o Royal Botanic Gardens Kew deixa de ser unicamente um jardim real e torna-se um dos maiores receptáculos de espécies vegetais, vindas de um número cada vez maior de regiões anexadas ao império britânico, julga-se imprescindível a edificação de uma palm house, cujos tamanho e “nobre estrutura” deveriam ser sem precedentes na Europa. O projeto, confiado a Decimus Burton, foi concluído em 1848 e apresentou um “novo sistema de vigas de ferro arqueadas” (Drayton, 2000, p.185) em um edifício de 110 metros de extensão, trinta metros de largura e vinte metros de altura, cuja área central contava ainda com uma espécie de mezanino a uma altura de nove metros do chão, que se chegava por meio de escadas em espiral, possibilitando com isso ao visitante ver a copa das palmeiras, mesmo as mais elevadas (Seemann, 1856, p.38-39).

O interior da palm house do Kew Gardens foi exibido por Berthold Seemann em seu livro sobre as palmeiras por meio de uma imagem por ele nomeada “A grande palm house no Jardim Real, Kew”. Na ilustração, é possível perceber a enorme dimensão do edifício com paredes e tetos de vidro, sustentados por estruturas e colunas feitas de ferro. Nota-se também uma profusa vegetação, admirada por casais, crianças e senhoras elegantemente vestidos (Figura 3).

**Figura 3:** : A grande palm house no Jardim Real, Kew (Seemann, 1856).

Tal fascínio também era ressaltado por Seemann nas páginas de seu livro. Segundo ele, o visitante experimentava naquele local a sensação de “repentinamente” encontrar a si mesmo em meio a uma densa vegetação tropical composta por bananeiras, bambus, samambaias, estrelícias, cactos, baunilhas, tamarindos, e uma “notável” coleção de palmeiras, que ele assim descrevia:

As duas palmeiras mais altas que prendem a atenção são as espécies de coqueiro (Cocos plumosa e Cocos coronata), ambas bons exemplares do extenso grupo com folhas penatissectas; as duas mais robustas, uma espécie de Sabal (S. umbraculifera), igualmente bons exemplares de outro grupo menos numeroso, distinguem-se pelas folhas em leque. Há ainda nesta coleção: − a tamareira (Phoenix dactylifera), produtora de frutos para o comércio e escritura; a palmeira palmyra (Borassus flabelliformis), uma das palmeiras mais difíceis de cultivar; o dendezeiro (Elaeis guineensis), que produz óleo de dendê; o coqueiro (Cocos nucifera), cujos usos são considerados mais numerosos que os dias do ano; a palmeira americana (Oreodoxa oleracea), cujas folhas jovens são um excelente vegetal suculento; a árvore noz de bétel (Areca catechu); a tamareira selvagem da Índia (Phoenix sylvestris), fornecedora de vinho de palma e açúcar; a planta-marfim (Phytelephas macrocarpa), cujas sementes assemelham-se à aparência do marfim animal; a palmeira de cera dos Andes (Ceroxylon andicola), cujo caule adulto é coberto por uma substância cerosa; e, por último, a palmeira vassoura (Attalea funifera), cuja fibra grossa é utilizada na confecção de vassouras e escovas (Seemann, 1856, p.40-41).^3^

Em seu relato, Seemann permite-nos saber quais espécies de palmeiras compunham a coleção do Kew Gardens, em 1856, aludindo – como autor de um livro destinado ao estudo e à descrição das palmeiras – sobre seus usos, sua morfologia, e a proveniência de cada espécie. Outras narrativas menos amparadas pelo olhar cientificista também nos ajudam, a seu modo, a entender o arrebatamento que as palmeiras ali reunidas causavam no espectador. D. Pedro V, o jovem rei de Portugal e Algarves, por exemplo, que, em 1854, visitou a palm house do Kew Gardens, na companhia do príncipe Albert, sem muitas reservas expressou em seu relato de viagem toda sua admiração pelas dimensões da edificação, cuja altura nem mesmo um “Cocos plumoso quase perfeitamente desenvolvido” (D. Pedro V, 1923, p.150) chegava a tocar o teto. Sobre a coleção em si, D. Pedro V convidava o leitor para um passeio imaginário e exibia seu maravilhamento pelas espécies coletadas em diferentes partes do mundo. Dizia ele:

Como descreverei a bela coleção de palmeiras?! É quase impossível. Imagine-se um vasto bosque de Latanias, Coryphas, Sabal, Chamaerops, Cynosureae, Encephalartos, formando os grupos mais pitorescos e dominado por Cocos, por Caryotas … A pena é insuficiente para retratar as belezas da natureza. Muito menos se pode descrever a impressão que causa à vista o voo de pássaro que se goza das varandas das estufas. Paira-se com o pensamento sobre a Ásia, a África, a América e a Oceania e também se pensa na Europa, porque só nessa se veem tão belas coisas reunidas (D. Pedro V, 1923, p.150).

Se as grandes estufas aquecidas eram indispensáveis para as coleções reais localizadas em países do Norte da Europa, em Portugal, diferentemente, tendo a vantagem de um inverno mais ameno, testemunhou-se o crescimento a céu aberto de um grande número de vários gêneros e espécies de palmeiras, sob o constante cuidado de D. Fernando II − rei consorte de D. Maria II e pai de D. Pedro V −, cujo interesse por jardins e horticultura o deixou conhecido, posteriormente, como “rei paisagista”. Com a ajuda de seu fiel jardineiro, Jean Baptiste Bonnard, D. Fernando II dedicou, ao longo de toda sua vida, imenso tempo e dinheiro na constituição do jardim do Palácio da Pena, residência real de verão, e da Tapada das Necessidades, localizada na região de Alcântara, em Lisboa. Nas palavras da historiadora Cristina Castel-Branco (2001, p.87), “D. Fernando escolhe para a Pena uma vegetação menos exuberante que para as Necessidades, na qual as palmeiras são a grande novidade”. Graças a uma visita do botânico alemão Edmond Goeze ao jardim das Necessidades, relatada em pormenor em um artigo publicado no Jornal de Horticultura Prática, do Porto, em 1876, pode-se vislumbrar os gêneros e espécies que, naquela época, faziam parte da coleção real de D. Fernando II.

Mesmo sendo a Tapada das Necessidades um espaço que congregava grande variedade de bambus, estrelícias, dracenas, araucárias, laranjeiras, limoeiros, figueiras, magnólias, entre outras, Goeze (1876, p.43) justificava começar sua narrativa pelas palmeiras, já que eram elas as “rainhas do reino vegetal”. Primeiramente, o botânico mencionava “a célebre palmeira coquita do Chile (Jubaea spectabilis)”, adquirida por D. Fernando há 18 anos, que ali havia se desenvolvido de uma maneira “espantosa”, contando naquele momento com dez metros de altura e quatro metros de circunferência, o que fazia dela “talvez a mais bela desta espécie em toda a Europa” (p.43). Em seguida, Goeze citava a recente aquisição de uma Livistona sinensis, para ele, uma “bela amostra chinesa”, que competia em beleza com a “robusta árvore da espécie Sabal adansonii”, que, por sua vez, já havia dado fruto com boas sementes para reprodução. O botânico alemão também comentava sobre uma Livistona australis (Corypha australis) e uma Seaforthia elegans, ambas da Austrália, cujos tamanho e beleza raramente eram vistos mesmo “nas maiores coleções da Europa” (p.43).

Goeze (1876, p.43) continuava seu passeio apontando, ainda, para um grupo de trinta Chamaerops, constituído por quatro espécies diferentes, “dispostas artisticamente”, formando, assim, o “non plus ultra da perfeição”. Mencionava igualmente uma Phoenix reclinata, que se achava ali “bem representada”; uma Phoenix dactylifera, em suas palavras, a espécie “mais vulgarizada em Portugal”, “onde às vezes ganha grande altura”; e também uma Cocos australis, para ele, a “mais majestosa” palmeira das Necessidades, alcançando de 12 e 15 metros de altura (Goeze, 1876, p.44).

A fascinação pelas palmeiras em solo europeu deu vez à construção de diversas outras palm houses em países como Inglaterra, Alemanha, Áustria, Dinamarca, Escócia e França. Embora não fossem necessárias estufas envidraçadas para o seu acolhimento, no Brasil, as palmeiras também simbolizaram, entre outros aspectos, nobreza e poder. Como se sabe, o próprio D. João VI, então príncipe regente, recém-chegado ao país, com suas próprias mãos plantou, em 1809, no Jardim Botânico do Rio de Janeiro, há pouco inaugurado por ele, uma Roystonea oleracea, originária das Antilhas, que passou a ser conhecida como palmeira-imperial. Com o tempo, esse tipo de palmeira começou a ser vista também em praças públicas, nos jardins de instituições renomadas, bem como nas residências de famílias abastadas, exibindo, como se acreditava, ares de distinção e nobiliarquia (D’Elboux, 2006, p.199).

## Considerações finais

Desde a repercussão dos escritos de Humboldt, no início do século XIX, torna-se perceptível um arrebatamento diante das palmeiras. Elas foram celebradas por seu formato “majestoso” em conjunção com suas múltiplas utilidades por diversos homens de ciência advindos, especialmente, dos estudos da botânica e horticultura. Mesmo levando em consideração as diferentes trajetórias e perspectivas intelectuais desses autores, nota-se um viés comum que discorreu sobre essa família de plantas, salientando seu lugar de primazia no imenso reino vegetal dos trópicos, algo que contribuiu significativamente para o enraizamento de uma experiência identitária de contraste. De um lado, uma região eminentemente retratada por sua natureza exuberante, cujos habitantes eram, em grande parte dos relatos, vistos como indolentes e selvagens, e do lado oposto, uma Europa, de clima temperado, modelo de progresso e civilização.

Pode-se dizer, com isso, que, em paralelo à emergência de um conjunto discursivo que inventou o Oriente como um espaço exótico, mítico e fantasioso (Said, 1996), foram produzidas também narrativas que definiram os trópicos a partir de sua natureza descrita como exuberante, soberba e espetacular. Os estudos da tropicalidade, nesse sentido, enfatizaram a presença dessa tônica discursiva que selou no imaginário europeu a presença de uma região dominada por sua natureza. Nesse arcabouço diversificado de textos e imagens, formado também pelas publicações de horticultura e botânica, as palmeiras receberam especial atenção, sendo descritas a partir de imagens e recursos linguísticos caros ao imaginário imperial europeu da época.

As palm houses, assim, podem ser vistas como símbolos privilegiados de poder e soberania. Isso porque sua gigantesca e moderna arquitetura exibia riqueza, progresso, mas, ao mesmo tempo, o domínio europeu sobre os trópicos. Afinal, sob seu teto estavam, além de uma variedade de outras plantas, as palmeiras, vistas como a própria majestade daquele distante, fantasioso e imenso reino vegetal.

## Data Availability

Não estão em repositório digital.

## NOTAS

1 A Maximiliana regia, conhecida popularmente como inajá, recebeu também outros nomes científicos como, por exemplo, Attalea maripa (Aubl.) Mart. (Lorenzi, 2010, p.87).

2 Nessa citação, as espécies foram mencionadas de acordo com o texto original e as medidas convertidas de pés para centímetro ou metro.

3 Nessa citação, manteve-se o nome popular das palmeiras mencionado pelo autor na versão em inglês.
